# Genetically Engineered Goats as Efficient Mammary Gland Bioreactors for Production of Recombinant Human Neutrophil Peptide 1 Using CRISPR/Cas9

**DOI:** 10.3390/biology13060367

**Published:** 2024-05-23

**Authors:** Dongxu Li, Rihong Guo, Fang Chen, Jingang Wang, Feng Wang, Yongjie Wan

**Affiliations:** 1 Jiangsu Livestock Embryo Engineering Laboratory, College of Animal Science and Technology, Nanjing Agricultural University, Nanjing 210095, China; 2022205013@stu.njau.edu.cn (D.L.); wangjingang73@163.com (J.W.); 2 Jiangsu Provincial Engineering Research Center for Precision Animal Breeding, Nanjing 210014, China; rhguo@jaas.ac.cn (R.G.); fchen_m@sina.com (F.C.); 3 Institute of Animal Science, Jiangsu Academy of Agricultural Sciences, Nanjing 210014, China

**Keywords:** CRISPR/Cas9, goat, HNP1, β-casein, antibacterial activity

## Abstract

**Simple Summary:**

Mammary bioreactors represent a promising method for the production of recombinant proteins. In this study, the human neutrophil peptide 1 (HNP1) sequence was knocked into the seventh exon of the goat β-casein (CSN2) gene under the control of the CSN2 promoter using CRISPR/Cas9 technology. A mixture of Cas9 mRNA, sgRNA, and a homologous plasmid including the T2A-HNP1 sequences were microinjected into the embryos of donor goat and then transplanted into recipient goats through embryo transfer technology. This allowed the HNP1 gene to be expressed in the offspring, facilitating the production of antimicrobial peptide proteins through the goat’s mammary glands. This experiment successfully produced genetically edited goats that secrete HNP1 with mammary-specific characteristics, providing a scientific basis for the further promotion of gene-editing technology and the development of new transgenic goat breeds with antimicrobial components in their milk.

**Abstract:**

Mammary gland bioreactors are promising methods for recombinant protein production. Human neutrophil peptide 1 (HNP1) exhibits antibacterial and immune-modulating properties. This study aims to establish a method to generate goats secreting HNP1 using the mammary gland as bioreactors. HNP1 transgenic goats were generated by using CRISPR/Cas9 technology to knock-in (KI) the HNP1 sequence into exon 7 of the goat β-casein (CSN2) gene under the control of the CSN2 promoter. One-cell stage embryos were cytoplasmically injected with a mixture of Cas9 mRNA, sgRNA, and a homologous plasmid including the T2A-HNP1 sequences, followed by transfer to recipient goats. A total of 22 live offspring goats were delivered, and 21 of these goats (95.45%) exhibited targeted edits at the *CSN2* locus, and 2 female goats (9.09%) demonstrated successful HNP1 integration. Western blot and ELISA analyses confirmed the presence of HNP1 protein at high levels in the milk of these HNP1-positive goats, with mean concentrations of 22.10 µg/mL and 0.0092 µg/mL during the initial 60 days of lactation. Furthermore, milk from these transgenic goats exhibited notable antibacterial activity against Escherichia coli and Staphylococcus aureus, demonstrating the functionality of the expressed HNP1 protein. In conclusion, we established an efficient method for developing new transgenic goat lines as a mammary gland bioreactor, and the bioactive HNP1 protein secreted by the transgenic goat has the potential to combat microbial resistance.

## 1. Introduction

The mammary gland bioreactor represents a highly efficient and economically viable method for the large-scale production of recombinant proteins [1]. Over recent years, significant advancements have been made in expressing various bioactive proteins within different animal mammary glands, including lactoferrin [2,3], human parathyroid hormone [4], α-fetoprotein [5], lysozyme [6], and butyrylcholinesterase [7]. Notably, ATryn stands as a groundbreaking example, being the first recombinant therapeutic protein drug produced using transgenic animals [8]. Approved by both the European Medicines Agency and the FDA, Atryn, produced in transgenic goats, demonstrates the potential of this approach. For instance, these goats can yield as much antithrombin in a year as 90,000 blood donations [9]. It is evident that the use of transgenic animals as mammary gland bioreactors holds tremendous promise for drug production. However, traditional methods for generating transgenic animals, such as somatic cell nuclear transfer (SCNT), have limitations in efficiency and labor intensity. The advent of gene editing technologies, particularly the CRISPR/Cas9 system, offers a more efficient pathway for the generation of transgenic livestock as mammary gland bioreactors.

The CRISPR/Cas9 system, derived from the Immune defense mechanisms of archaea and bacteria, has revolutionized gene editing [10]. This technology employs engineered single guide RNAs (sgRNAs) to direct the Cas9 protein to specific genomic sequences, enabling precise DNA cleavage at desired locations [11,12]. Compared to earlier gene editing tools like zinc finger nucleases (ZFNs) and transcription activator-like effector nucleases (TALENs), CRISPR/Cas9 offers unparalleled advantages in terms of simplicity, cost-effectiveness, and editing efficiency [13,14]. Following DNA cleavage, two main repair pathways, non-homologous end joining (NHEJ) and homology-directed repair (HDR), are activated. While NHEJ can result in unpredictable insertions or deletions, HDR allows for precise sequence insertion, provided a foreign DNA donor template is available [14]. This versatility enables precise gene knock-in (KI) or knock-out (KO), making CRISPR/Cas9 indispensable for the development of genetically modified animal models and production lines. For example, using CRISPR/Cas9 and cytoplasm injection of one-cell stage embryo, *MSTN* KO goats [15,16], sheep [17,18], rabbits [15,19,20], and pigs [21] with double muscling phenotype have been efficiently generated. While the experience in generating transgenic livestock using cytoplasmic injection of the Cas9 system to zygotes is still limited, further work should be carried out to test the feasibility of the method.

Antimicrobial peptides (AMPs), comprising 12 to 100 amino acids, exhibit antimicrobial activity against a wide range of pathogens [22,23,24]. These molecules play a crucial role in the innate immune system, serving as the first line of defense against invading pathogens. As AMPs mainly target microbial membranes, impeding the ability of microbes to develop resistance against them, which makes them promising candidates for new antibiotics [22]. Human neutrophil peptide 1 (HNP1), a specific α-defensin composed of 30 amino acids, is the main defensin in human neutrophils [25]. However, the clinical application of HNP1 has been hindered by challenges in mass production [26]. Methods such as extraction from human peripheral blood, chemical synthesis, and bioreactor expression have been explored for HNP1 production. The yield of mature HNP1 from human peripheral blood is severely limited, and the cost of chemically synthesizing this peptide is prohibitively high for large-scale production. Consequently, significant efforts have been dedicated to utilizing prokaryotic systems to express mature HNP1 directly [26]. Previous attempts to employ prokaryotic systems for the direct expression of mature HNP1 have encountered issues such as cytotoxicity to host cells and challenges in achieving proper folding within a short time [27,28]. Various fusion protein strategies have been implemented to mitigate the toxicity and enhance solubility of mature HNP1, yet with suboptimal outcomes, possibly due to the peptide’s folding intricacies and persistent toxic effects. Consequently, the development of a feasible method for the large-scale and cost-effective production of mature HNP1 has not yet been achieved. Addressing this challenge, the use of mammary gland bioreactors presents a promising strategy for the scalable production of mature HNP1, potentially overcoming current limitations in production efficiency and cost.

**Figure 1:**
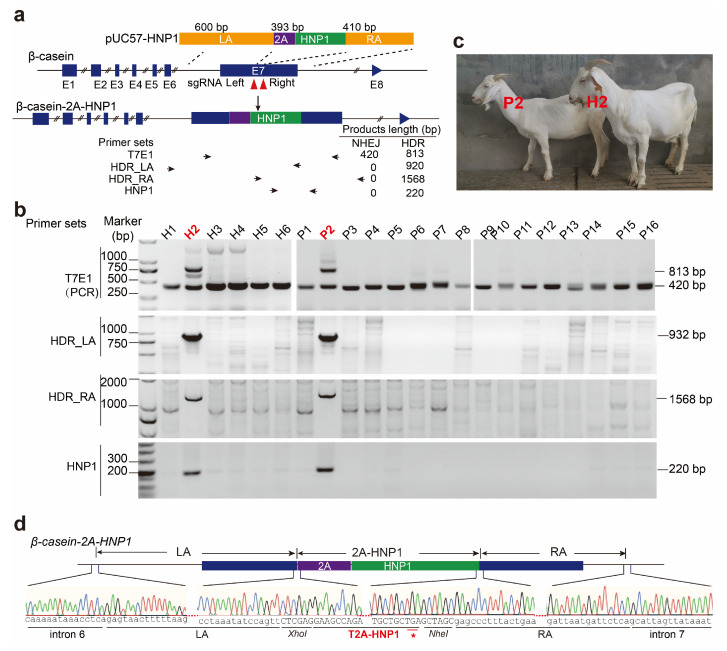
Cas9-mediated HNP1 knock-in in goats at the *CSN2* locus: (**a**) Schematic diagram of experiment design for HNP1 insertion into goat CSN2. sgRNA left and right were designed to target exon 7 of CSN2. The plasmid PUC57-HNP1 with the left arm (LA), 2A-HNP1, and the right arm (RA) was used as a homologous repair template for HNP1 insertion. Four primer sets were utilized for genome editing detection. In wild type (WT) and CSN2 KO goats (repaired through the NHEJ method), only a 420 bp fragment could be amplified using the T7E1 primer sets. In HNP1-inserted goats (repaired through HDR method), an additional 813 bp fragment could be amplified. The other three primer sets, HDR_LA, HDR_RA, and HNP1, only amplified the expected fragments in HNP1-inserted goats. (**b**) PCR analysis using the four primer sets. (**c**) The two goats (H2 and P2) with HNP1 insertion at *CSN2* locus. (**d**) TA clone sequencing results of HDR_LA and HDR_RA amplicons. * following the T2A-HNP1, indicating the stop codon.

In this study, we employed CRISPR/Cas9 technology to insert the HNP1 sequence into exon 7 of the goat β-casein (CSN2) gene (Figure 1a). Leveraging the CSN2 promoter, this strategy enables the specific expression of HNP1 within the mammary gland, effectively converting it into a bioreactor for producing the antimicrobial peptide. Our research not only demonstrates the feasibility of generating HNP1-expressing goats but also lays the groundwork for the development of novel, high-quality dairy products through gene editing technology in goats. Furthermore, it highlights the potential of CRISPR/Cas9 as a valuable tool for genetic modification in this species.

## 2. Materials & Methods

### 2.1. Ethics Statement

All the hormones or analogs used in this study were provided by Ningbo Sansheng Biological Technology Co., Ltd. (Ningbo, China), with experimental consumables supplied by Guangzhou Jet Bio-Filtration Co. (Guangzhou, China), unless otherwise mentioned. The animal experimental protocol of this study was approved by the Animal Care and Use Committee of Nanjing Agricultural University.

### 2.2. Plasmid Construction and In Vitro transcription

Two sgRNAs, sgRNA-left (AAGGGCTCAACTGGATATTT) and sgRNA-right (CATCAGTGAGAGTCAGGCTC), targeting exon 7 of goat CSN2 were selected. The oligos for each sgRNA (Table S1) were annealed and cloned into the pX330 plasmid, which contains a Cas9 expression cassette and an sgRNA cassette. The in vitro transcription templates for Cas9 and sgRNAs were amplified using the T7 promotor-attached primers (Table S1) and gel-purified using QiaQuick Spin Column (Qiagen, 28115, Venlo, The Netherlands). The Cas9 template was subjected to the T7 Ultra Kit (Ambion, AM1345, Naugatuck, CT, USA) and the sgRNA templates were transcribed in vitro using MEGA shortscript Kit (Ambion, AM1354). All Cas9 mRNA and sgRNAs were purified using the MEGA clear Kit (Ambion, AM1908).

A 1393 bp DNA fragment containing the 600 bp LA, 393 bp T2A-HNP1 (including stop codon), and 400 bp RA was synthesized and inserted between the XhoI and NheI restriction sites of pUC57 plasmid to construct the PUC57-HNP1 plasmid (Supplementary txt).

### 2.3. Preparation and Injection of One-Cell Embryos

Healthy goats (2–3 years old) were housed at the Haimen Goat Industry Research Institute in Nantong, Jiangsu. Goats were treated using established methods [15]. Briefly, a progesterone sponge was implanted in the goat’s vagina for 11 days. Following removal, the animals received 100 IU of PG. Donors were treated with a total of 200 IU FSH twice daily in a decreasing dose regimen (50/50, 25/25, and 25/25 IU) over 3 days, starting 48 h before sponge removal. Mating occurred at 36 h and 48 h after sponge removal. Recipients received 100 IU PMSG 24 h before sponge removal. One-cell stage embryos were flushed from the donor oviducts 72 h after sponge removal.

Harvested embryos were then injected with a mixture of 200 ng/μL Cas9 mRNA, 50 ng/μL sgRNA-left, 50 ng/μL sgRNA, and 50 ng/μL HNP1 plasmid. Following injection, the embryos were cultured in M2 media (Sigma, M7167, Kawasaki, Japan) containing 10% FBS (Gibco, 26140, Billings, MT, USA) in a 38.5 °C, saturated humidity, and 5% CO_2_ incubator. The cleaved embryos at the two-cell stage to blastocyst stage were transferred into 32 estrous-synchronized recipient goats. Early pregnancy was confirmed by ultrasound 30–45 days after embryo transfer.

### 2.4. Genome Editing Analysis

Genomic DNA was extracted from ear tissue of the offspring goats using a DNA extraction kit (Tiangen, DP348, Beijing, China). Four primer sets (Table S2) were used for genome editing detection. The primer set T7E1 was used to amplify exon 7 of CSN2, and only goats with HNP1 insertion would have an additional band larger than the 420 bp products. The other three primer sets HDR-LA, HDR-RA, and HNP1 with forward or (and) reverse primer cross HNP1 could only amplify the products with expected size in HNP1-inserted goats. The regions flanking each target site were amplified by PCR using PrimerStar HS DNA polymerase (Takara, R010A, San Jose, CA, USA). The PCR products were purified using a QiaQuick spin column (Qiagen, 28115, Venlo, The Netherlands) following the manufacturer’s instructions. The PCR products were then subcloned into the pMD-19T vector (Takara 3271, San Jose, CA, USA). For each sample, 12 to 25 random colonies were selected for Sanger sequencing.

### 2.5. Western Blot Analysis

The edited female goats were mated after reaching sexual maturity, and their milk was collected every 10 days after delivery for 60–70 days until weaning. The whey protein was separated according to the previous studies [29]. Briefly, the milk samples were centrifuged at 3000× *g* for 15 min, and the upper fat fractions and the lower sediment fractions were removed. The middle fluids were then adjusted to pH = 4 with 1M HCl and centrifuged at 3000 rpm for 20 min to eliminate the casein fraction. Then, the protein samples were denatured in gel loading buffer at 95 °C for 10 min. Denatured proteins were separated by 15% Tricine-SDS-PAGE and transferred to polyvinylidene fluoride (PVDF) membranes (Millipore, 03010040001, Billerica, MA, USA). The membranes were blocked with 5% BSA for 1 h at room temperature, followed by incubation with primary antibodies (Affinity Biosciences, DF8527, Cincinnati, OH, USA) at 4 °C for 12–16 h. After three washes with TBST (Tris-buffered saline containing 0.1% Tween 20), the membranes were incubated with the secondary antibody (ThermoFisher Scientific, 31460, Waltham, MA, USA) for 1 h at room temperature and visualized using Image Quant LAS 4000 (Fijifilm, Tokyo, Japan).

### 2.6. Enzyme-Linked Immunosorbent Assay (ELISA) Analysis

The concentration of HNP1 in the milk samples was determined using an ELISA kit (Boster, EK1514, Pleasanton, CA, USA). Briefly, diluted standards and whey protein samples were incubated in a 96-well plate at 37 °C for 90 min. Following incubation, a biotin-labeled antibody specific to HNP1, diluted 1:100, was added and incubated for an additional 60 min at 37 °C. The wells were then washed thoroughly with a washing buffer, followed by incubation with an enzyme conjugate (ABC solution) diluted 1:100, at 37 °C for 30 min. After further washing, the plate was incubated in the dark for 22 min before adding a termination liquid. The optical density (OD) of each well was measured using an enzyme labeling instrument (Thermo, Multiskan FC, San Jose, CA, USA), and the HNP1 concentration in the whey protein samples was calculated using ELISACalc software (version 0.2). The detection range of the kit was 321~20,000 pg/mL.

### 2.7. Antibacterial Activity Analysis

Antibacterial activity of the HNP1-enriched milk was assessed using a disc diffusion assay. Freeze-dried milk powder was diluted and loaded onto 6 mm diameter filter paper discs, creating drug-sensitive papers. These papers were then exposed to suspensions of Escherichia coli (ATCC25922) and Staphylococcus aureus bacteria (ATCC25923) to mimic potential pathogens. The experiment was conducted in controlled laboratory conditions with the papers placed in culture medium and incubated at 37 °C. The resulting zones of bacterial inhibition around the discs were observed and measured to quantify the HNP1 protein’s effectiveness in suppressing bacterial growth.

## 3. Results

### 3.1. Generation of HNP1-Expressing Goats

The process of generating HNP1 protein is outlined in Table 1. Ninety-six zygotes retrieved from donor female goats were microinjected with a mixture of Cas9 mRNA, sgRNA-right, and sgRNA-left targeting exon 7 of the *CSN2* locus and the donor plasmid PUC57-HNP1 (Figure 1). Out of these, seventy-four embryos developed normally and were subsequently transferred to 32 recipient goats. Pregnancy was confirmed by ultrasound at 45 days in 59.38% (19 out of 32) recipients. Following full gestation, 23 offspring were delivered, with one stillbirth.

**Figure 2:**
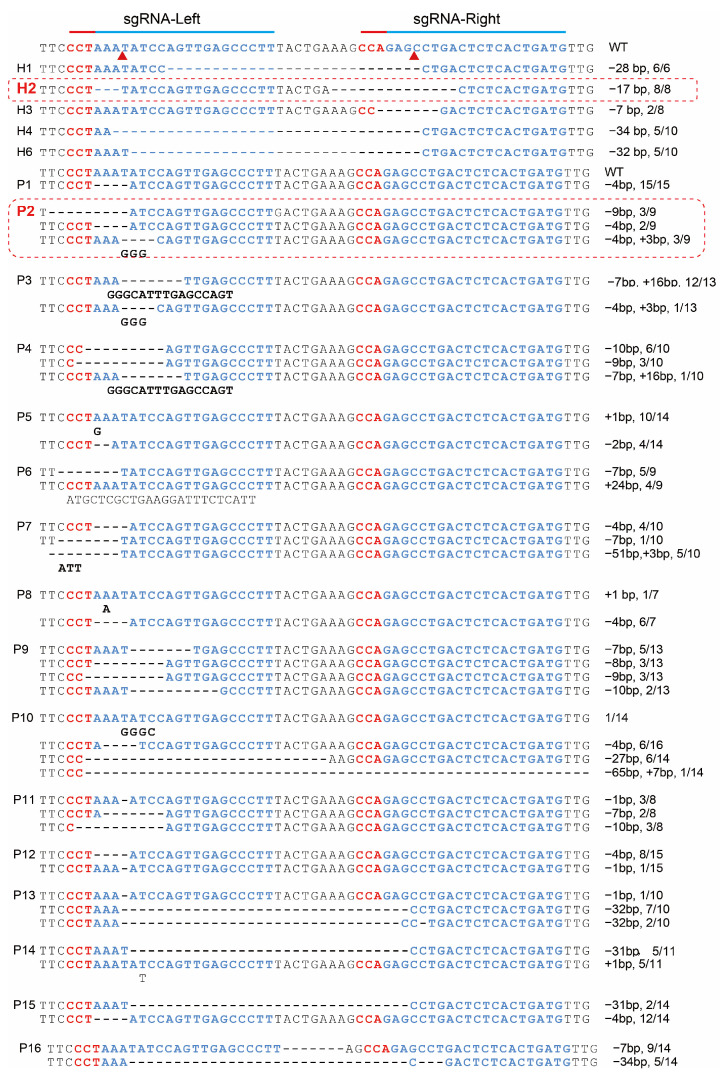
TA clone sequencing results of goat *CSN2* locus. “−” in the sequences indicates deletions; bases under the sequences indicate insertions; “−” and “+” immediately behind each sequence indicate deletion and insertion, respectively. The ratio represents the proportion of mutated clones out of the total clones sequenced. WT, wild-type; 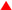 indicating the cas9 cutting site.

PCR analysis using T7E1 primers in two female goats (H2 and P2) revealed additional amplicons of approximately 813 bp alongside the expected 420 bp fragments, indicating successful HNP1 gene integration into the *CSN2* locus. TA cloning and subsequent sequencing identified mutations ranging from 53 bp deletions to 16 bp insertions in the targeted region of 21 (95.45%) offspring (Figure 2). Over more, 16 goats (H1, H2, P1~P11, P13, P15, P16) were totally edited, while in H3 H4, H6, P12, and P14, 25%, 50%, 50%, 75%, and 90.91% of CSN2 clones were edited. Consistent with the results obtained using T7E1 primers, PCR amplification using the primer sets HDR-LA, HNP1, and HDR-RA confirmed HNP1 integration at the intended β-casein location in goats H2 and P2 (Figure 1b,d). TA cloning and subsequent sequencing of amplicons of HDR-LA and HDR-RA primer sets indicated precise HNP1 insertion into the *CSN2* locus at both 5’ and 3’ sides of both H2 and P2 (Figure 1d). These results indicated that non-homologous end joining (NHEJ) occurred in 95.45% goats and homologous recombination directed repair (HDR) occurred in 9.09% (2 out of 22) of the offspring.

### 3.2. Production of HNP1 in Milk

**Figure 3:**
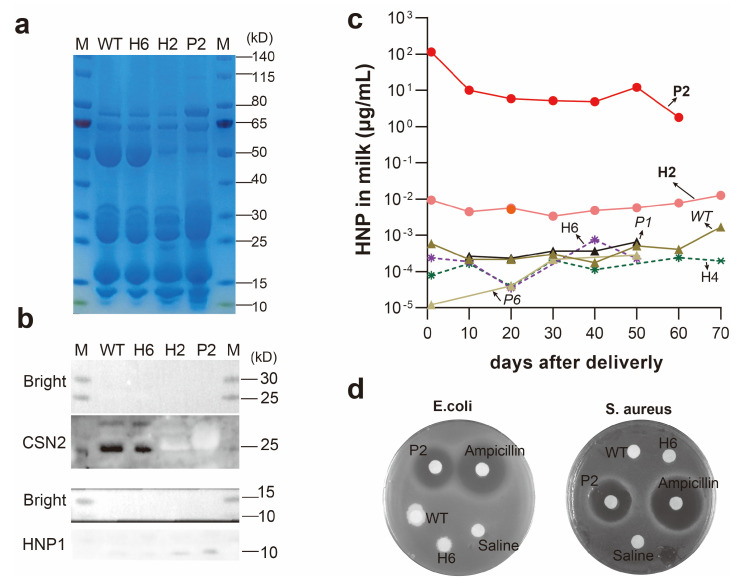
Protein content and antimicrobial activity analysis in goat milk. (**a**) Coomassie staining of goat milk protein. (**b**) Detection of HNP1 and CSN2 protein in whey protein by Western blot. (**c**) Quantification of HNP1 protein content in whey protein using ELISA. (**d**) Assessment of antimicrobial activity of HNP1 protein against Escherichia coli and Staphylococcus aureus. P2 and H2, HNP1-inserted goats; P1, P6, H4, and H6, CSN2 indels goats; WT, wild-type goats.

After reaching sexual maturity, founder females were mated. Milk samples were collected every 10 days postpartum for the analysis of HNP1 content using Coomassie staining (Figure 3a), Western blot (Figure 3b), and ELISA (Figure 3c). Western blot analysis confirmed that the CSN2 protein was only expressed in wild-type (WT) and CSN2 indels from goat milk samples of H6, while CSN2 expression was not detected in the milk of goats H2, P2, and CSN2 bi-allelic indels from goat milk samples of H1 (Figure S1); HNP1 protein was only present in the milk of goats H2 and P2, with no detectable HNP1 expression in WT or no HNP1 KI goats (Figure 3b).

### 3.3. Quantification of HNP1 Levels

Further ELISA analysis revealed high levels of HNP1 in the milk of the goats that produced it (H2 and P2) during the first 60 days after giving birth (lactation). The average concentration of HNP1 in the milk of goat P2 was 22.10 µg/mL, while the milk of goat H2 contained an average concentration of 0.0092 µg/mL (Figure 3c). In contrast, the levels of HNP1 in the milk of regular goats and those genetically modified not to produce HNP1 remained below 1000 pg/mL. Given that these concentrations approached the kit’s lower detection limit of 325 pg/mL, they are likely attributable to the inherent background levels in milk.

### 3.4. Antibacterial Activity of HNP1 Milk

To determine the functionality of the HNP1 protein in milk, its ability to inhibit the growth of Escherichia coli and Staphylococcus aureus was evaluated. Milk from goat P2, a genetically modified positive goat, displayed a clear area around it where neither E. coli nor S. aureus bacteria could grow (bacteriostatic zone). This zone of inhibited bacterial growth was similar to the area of inhibition observed around a positive control containing ampicillin, an antibiotic (Figure 3d). In contrast, no zone of inhibited bacterial growth was observed around milk from regular goats, sterile water, or milk from H6, a genetically modified goat without HNP1 integration. These findings confirm that the genetic modification process successfully produced functional HNP1 in the milk of the modified goats.

## 4. Discussion

Mammary gland bioreactors are promising methods for recombinant protein production. ATryn, produced in transgenic goats, is the first recombinant therapeutic protein drug approved by both the European Medicines Agency and the FDA [8]. However, generation of KI animals are always based on HR and SCNT in previous studies, which has low efficiency and is lab-intensive. In this study, HNP1 KI goats were efficiently generated as mammary gland bioreactors by cytoplasmic injection of Cas9 systems into goat zygotes. Furthermore, this is the first report to express bioactive HNP1 protein using mammary gland bioreactors.

Guided with two sgRNAs, the genome editing occurred in 95.45% of the newborn goats with 68.18% of goats totally edited at the *CSN2* locus in this study. The high genome editing efficiencies were in accordance with our previous results in rabbits [20] and sheep [17]. To be noted, we even achieved total MSTN KO in 90% of the edited sheep using four sgRNAs, resulting in typical double muscling phenotype in the founder sheep [17]. These results provide a method for high gene KO, which is also important for designed breeding for livestock.

Traditional production for transgenic animal is always based on screening of the KI cell clones with antibiotics and SCNT. Both steps are lab-intensive and have low efficiency; the gene integration is also random. Moreover, the selection markers may do harm to the founder health, and the cloned animals are always with health problems. Using cytoplasmic injection with Cas9 system and donor plasmid, we achieved 9.09% HNP1 integration at the *CSN2* locus. This KI rate is relatively low compared to the KO rates. It could be easily understood as NHEJ is the dominant genome repair pathway after DSB is introduced to the target site.

As our study was carried out, rapid advancements in genome editing technologies have led to the development of several novel gene KI methods [30], including Easi-CRISPR [31], Tild-CRISPR [32], and PASTE [33]. Easi-CRISPR and Tild-CRISPR utilized long single-stranded DNA (lssDNA) and linearized double-stranded donor DNA (dsDNA) as donor templates respectively, departing from the traditional circular DNA plasmid approach. Tild-CRISPR has demonstrated impressive integration rates, ranging from 6.9% to 54.4% in mouse embryos, for the insertion of cargoes ranging from 0.8 kb to 6.0 kb. Moreover, it has exhibited up to a 12-fold increase in KI efficiency in human embryos, showcasing its potential for clinical applications. Similarly, Easi-CRISPR has shown promising insertion efficiencies, ranging from 8.5% to 100% in newborn mice, employing lssDNA with 60–100 bp homologous arms and approximately 800 bp cargoes. Distinctively, PASTE represents a novel genome editing system that combines Prime editing with CRISPR-directed integrases. Notably, PASTE achieves precise and efficient integration of transgenes without the requirement for double-strand DNA cleavage, enabling the insertion of large DNA cargoes ranging from 779 bp to 36,000 bp. These innovative methods hold great promise for applications such as the generation of animals as mammary gland bioreactors.

The mammary gland bioreactor holds considerable potential for the efficient synthesis of recombinant proteins. Previous studies have demonstrated that transgenic mice can produce β-lactoglobulin (BLG) at levels up to 30 µg/mL in their milk. In a similar vein, transgenic goats and cows have been engineered to produce recombinant human lactoferrin (rhLF) at concentrations of 30 µg/mL and 13.6 µg/mL [34], respectively. In the current study, the milk from two transgenic goats contained HNP1 protein at concentrations of 22.10 µg/mL and 0.0092 µg/mL, indicating significant variability in expression. Past research using CSN promoters to drive exogenous drug protein expression in the mammary glands of transgenic goats and cattle generated through homologous recombination (HR) showed expression levels ranging from 9.5×10^−5^ to 14 mg/mL [35,36]. This wide range of expression could be due to differences in the foreign gene, copy number, integration sites, gene constructs, and epigenetic modifications [36]. In this study, HNP1 was integrated site-specifically into the goat *CSN2* locus. Therefore, of the factors listed, only epigenetic variations could potentially account for the differential expression between goats H2 and P2. The extent to which epigenetic modifications have affected HNP1 protein expression remains to be established. Notably, the milk yield of goat H2 was markedly lower than that of other goats, which suggests aberrations in the production of milk proteins, including those encoded by the CSN gene. This irregularity may also be responsible for the reduced HNP1 concentration observed in H2 compared to P2.

The production of HNP1 protein without fusion tags, folding requirements, and host cytotoxicity in the mammary gland bioreactor is a significant advancement compared to traditional prokaryotic systems. Given the prolonged lactation period of transgenic goats, the production of HNP1 is efficient and cost-effective. Future research will focus on optimizing the purification of HNP1 protein from milk and assessing its bioactivity in vivo.

## 5. Conclusions

In summary, we have demonstrated an efficient method to generate HNP1 KI goats, and use of these transgenic goats as bioreactors to produce bioactive HNP1, suggesting zygote cytoplasmic injection of Cas9 system and a donor plasmid can serve as a powerful method to generate transgenic animals as mammary gland bioreactors for the synthesis of functional pharmaceutical proteins. The study presented here develops a novel and robust approach for large-scale production of high-quality therapeutic proteins, which will be beneficial for both human health and biomedicine.

## Figures and Tables

**Table 1:** Summary of gene editing at goat *CSN2* locus.

Embryo No.	Recipient No.	Offspring No.			
Transferred/injected	Pregnant/total (%)	Live	CSN2 indels (%)	CSN2 bi-allelic indels (%)	HNP1 insertion (%)
74/92	19/32 (59.38)	22	21 (95.45)	16 (72.73)	2 (9.09)

## Data Availability

The data presented in this study are available on request from the corresponding author.

## Supplementary Materials

The following supporting information can be downloaded at: https://www.mdpi.com/article/10.3390/biology13060367/s1, Figure S1: Detection of HNP1 and CSN2 protein in whey protein by Western blot. Table S1: sgRNAs and primers for sgRNA cloning and in vitro transcription. Table S2: Primers for genotyping. Supplementary txt: LA-T2A-HNP1-RA sequence.
