# Supplementary material for: Genetically Engineered Goats as Efficient Mammary Gland Bioreactors for Production of Recombinant Human Neutrophil Peptide 1 Using CRISPR/Cas9
Source: Biology (Basel). 2024 May 23;13(6):367. doi: 10.3390/biology13060367 (PMC11200946; doi:10.3390/biology13060367)
Supplement: Supplementary file 1 [file biology-13-00367-s001.zip › biology-2990139-supplementary.pdf]

Figure S1

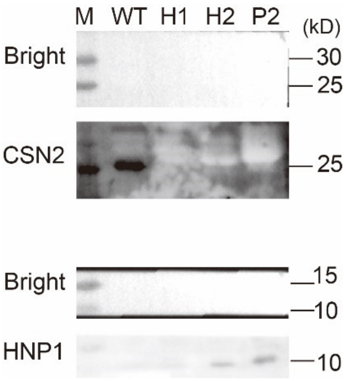

Detection of HNP1 and CSN2 Protein in whey protein by Western Blot. P2 and H2, HNP1 inserted goats; H1, CSN2 indels goats; WT, wild type goats.

Table S1 sgRNAs and primers for sgRNA cloning and in vitro transcription.

sgRNA	type	sequences
Left	sgRNA	AAGGGCTCAACTGGATATTTAGG
	F	cccagAAGGGCTCAACTGGATATTTAGG
	R	aaacAAATATCCAGTTGAGCCCTTc
	IVT_F	ttaatacgactcactatagggAAGGGCTCAACTGGATATTTAGG
Right	sgRNA	CATCAGTGAGAGTCAGGCTC
	F	caccgCATCAGTGAGAGTCAGGCTC
	R	aaacGAGCCTGACTCTCACTGATGc
	IVT_F	ttaatacgactcactatagggCATCAGTGAGAGTCAGGCTC
	IVT_R	AAAAGCACCGACTCGGTGCC

Table S2 Primers for genotyping.

Primer sets		Primer sequences	Product length (bp)	
			WT and β-CN KO	HNP Knockin
T7E1-PCR	F	AAAATCCACCCCTTTGCCCA	420	813
	R	GGCCTGGATGGGCATATCTC		
HNP	F	GCAGGAGAACGTCGCTATGG	0	220
	R	GGAGGAAACATGACGGTTGGA		
HDR_LA	F	CTACCACTCTGCAGGCAACT	0	932
	R	GGCTTTCAGTAAAGGGCTCG		
HDR_RA	F	GTGACAGTGTGGCACTAATCC	0	1568
	R	GCACGCTGGTATTCTGCAATAG		

Supplementary txt

LA-T2A-HNP1-RA sequence

GAGTAACTTTTTAAGTCTTTTTAAAATAGATCTTTCTTTGTTATATGAAATCAGTTTGGACTATTATCCAA
AGTATGTAGCTACCACTCTGCAGGCAACTCAGGAAGAGGTGGAATAAGTGTTGAAATCTCCAAACCCT

GATTTCACTTGACTCTCTGATTTCACTTGTGAAGGAAGTTGGGTTAATGAGAAATCCTTCAGCGAGCAT
TTTACTCATTAGTCTTCATATGACCCCAAACAATTTCTTAACTAAACCAAATGGAAGATTTTCTTTCTCT
CTCTTCACTGAATTATGTTTTAAAAAGAGGAGGATAATTCATCATGAATAACAATTATAACTGGATTATG
GACTCAAAGATTTTTTTTCCTTCTTTCCAGGATGAACTCCAGGATAAAATCCACCCCTTTGCCCAGGCA
CAGTCTCTAGTCTATCCCTTCACTGGGCCCATCCCTAACAGCCTCCCACAAAACATCCTGCCTCTTACT
CAAACCCCTGTGGTGGTGCCGCCTTTCCTTCAGCCTGAAATAATGGGAGTCCCCAAAGTGAAGGAGA
CTATGGTTCCTAAGCACAAAGAAATGCCCTTCCCTAAATATCCAGTTCTCGAGGAAGCCAGACACAAA
CAGAAAATTGTGGCACCGGTGAAACAGGAGGGCAGAGGAAGTCTTCTAACATGCGGTGACGTGGAG
GAGAATCCCGGCCCAATGAGGACCCTCGCCATCCTTGCTGCCATTCTCCTGGTGGCCCTGCAGGCCCA
GGCTGAGCCACTCCAGGCAAGAGCTGATGAGGTTGCTGCAGCCCCGGAGCAGATTGCAGCGGACATC
CCAGAAGTGGTTGTTTCCCTTGCATGGGACGAAAGCTTGGCTCCAAAGCATCCAGGCTCAAGGAAAA
ACATGGCCTGCTATTGCAGAATACCAGCGTGCATTGCAGGAGAACGTCGCTATGGAACCTGCATCTACC
AGGGAAGACTCTGGGCATTCTGCTGCTGA*GCTAGCGAGCCCTTTACTGAAAGCCAGAGCCTGACTCT
CACTGATGTTGAAAAGCTGCACCTTCCTCTGCCTCTGGTCCAGTCTTGGATGCACCAGCCTCCCCAGC
CTCTTTCTCCAACCGTCATGTTTCCTCCTCAGTCCGTGCTGTCCCTTTCTCAGCCCAAAGTTCTGCCTG
TTCCCCAGAAAGTAGTGCCCCAGAGAGATATGCCCATCCAGGCCTTTCTGCTGTACCAGGAGCCTGTA
CTTGGTCCTGTCCGGGGACCCTTCCCTATTCTTGTAAGTCTAAATTTACTAACTGTGCTGTTTAACTTCT
GATGTTTGTATGATATTTGAGTAATTAAGAGCCCTACAAAAAATCAATAATGAATGGTTCCAAAATAAGC
ATAGCTGAGATTAATGATTCTCA
